# A case–control study of pre-operative levels of serum neutrophil gelatinase-associated lipocalin and other potential inflammatory markers in colorectal cancer

**DOI:** 10.1186/1471-2407-14-912

**Published:** 2014-12-03

**Authors:** Laurence Duvillard, Pablo Ortega-Deballon, Abderrahmane Bourredjem, Marie-Lorraine Scherrer, Georges Mantion, Jean-Baptiste Delhorme, Sophie Deguelte-Lardière, Jean-Michel Petit, Claire Bonithon-Kopp, for the AGARIC study group

**Affiliations:** Inserm UMR 866, Faculté de médecine de Dijon, Dijon, 21079-F France; Biochemistry department, University hospital of Dijon, Dijon, 21070-F France; Department of digestive surgical oncology, University hospital of Dijon, Dijon, 21000-F France; Inserm CIC 1432, Faculté de médecine de Dijon, Dijon, 21079-F France; Clinical investigation center (team clinical epidemiology), University hospital of Dijon, Dijon, 21000-F France; Department of general and digestive surgery, Hôpital Brabois, University hospital of Nancy, Vandoeuvre-les-Nancy, Nancy, 54511-F France; Department of general, digestive and oncologic surgery, University hospital of Besançon, Besançon, 25030-F France; Department of general and digestive surgery, Hôpital de Hautepierre, University hospital of Strasbourg, Strasbourg, 67098-F France; Department of general, digestive and endocrine surgery, University hospital of Reims, Reims, 51100-F France; Biochimie Médicale, Plateau Technique de Biologie, 2, rue Angélique Ducoudray, BP 37013, 21070 Dijon Cedex, France

**Keywords:** Colorectal cancer, Case–control study, CRP, Inflammation, NGAL, NGAL/MMP-9, sTNFR-1, sTNFR-2

## Abstract

**Background:**

Chronic inflammation is a key feature of colorectal cancer (CRC), meaning that inflammatory biomarkers may be useful for its diagnosis. In particular, high neutrophil gelatinase-associated lipocalin (NGAL) expression has been reported in CRC. Thus, we investigated whether serum NGAL and NGAL/MMP-9 could be potential biomarkers for the early detection of CRC. Concurrently, we studied other inflammatory biomarkers such as soluble tumor necrosis factor receptor 1 and 2 (sTNFR-1, sTNFR-2), and C reactive protein (CRP).

**Methods:**

The AGARIC multicenter case–control study was performed in eastern France and included patients admitted for elective surgery either for *a priori* non-metastatic incident CRC (n = 224) or for benign causes (n = 252). Pre-operative serum levels of NGAL, NGAL/MMP-9, sTNFR-1, sTNFR-2 and CRP were measured.

**Results:**

Median values of serum NGAL, NGAL/MMP-9, sTNFR-1, sTNFR-2 and CRP were significantly higher in CRC patients than in controls. Receiver Operating Characteristic analysis provided relatively poor values of area under the curve, ranging from 0.65 to 0.58. Except for NGAL/MMP-9, all biological parameters were strongly correlated in CRC cases and, less strongly in controls. Multivariate odds ratio (OR) of CRC comparing the extreme tertiles of serum NGAL was 2.76 (95% confidence interval (CI): 1.59-4.78; p < 0.001),. Lower but significant multivariate associations were observed for sTNFR-1, and sTNFR-2: OR = 2.44 (95% CI : 1.34-4.45, p = 0.015) and 1.93 (95% : CI 1.12-3.31), respectively. No independent association was found between case–control status and NGAL/MMP-9. Among CRC cases, maximal tumor size was an independent determinant of serum NGAL (p = 0.028) but this association was reduced after adjustment for CRP (p = 0.11).

**Conclusion:**

Despite a significant increase in serum NGAL and other inflammatory markers among CRC patients, our findings suggest that they may not be suitable biomarkers for the diagnosis and especially early detection of CRC.

## Background

Colorectal cancer (CRC) is the second and third most common cancer in women and men, respectively, and the fourth leading cause of cancer-related death worldwide [1, 2]. Detection of CRC at an early stage is critical since the 5-year survival rate ranges from 96% for patients with stage I CRC to 5% for patients with stage IV CRC [3]. About half of CRCs are detected at advanced stages. Thus, the search for new markers is of major interest for early detection of CRC and identification of new potential therapeutic targets.

During recent years, Neutrophil Gelatinase-Associated Lipocalin (NGAL) gave rise to great interest in the oncology field. NGAL is a 198 amino acid glycoprotein first isolated in human neutrophils and expressed in many tissues [4]. In normal tissues, NGAL provides protection against bacterial infection and oxidative stress [5–8]. High NGAL expression was reported in many inflammatory benign diseases [9–16], as a consequence of a positive transcriptional regulation by inflammatory cytokines.

The diagnostic or prognostic potential of NGAL appears very variable according to the type of cancer. In tissues such as thyroid, breast, endometrium, pancreas, NGAL expression was shown to be null or weak in non-neoplastic tissues and to increase in the presence of dysplasia or neoplasia [17–23]. Moreover, some studies suggested that NGAL could be a predictor of disease-free survival in breast cancer [21, 22, 24]. Conversely, another report found a positive association between NGAL expression and the degree of differentiation of carcinoma cells in ovarian cancer [25].

Regarding serum NGAL concentration, several studies have reported higher levels in cancer patients than in healthy controls, for ovarian, gastric, pancreatic and kidney cancers [17, 18, 25, 26]. With respect to CRC, relatively few studies have investigated the role of NGAL in the development and progression of the neoplastic process. In CRC, immunohistochemical staining experiments demonstrated that NGAL expression in normal tissue was null or weak in 98% of cases, whereas it was moderate or intense in 74% of carcinoma. Moreover, a higher proportion of stages III and IV CRC expressed NGAL intensively compared to stages I and II (57% versus 42%) [27]. Small-sized studies even suggested that NGAL tissue expression could be a marker for poor prognosis in stage I CRC [28, 29]. Previous studies having examined serum NGAL levels provided discordant results possibly due to their small sample size and their inability to take into account potential confounders [27, 30]. Sun et al., did not show any significant increase in serum NGAL concentration in 39 CRC patients compared to matched controls, nor any significant association with cancer stage [27]. Fung et al. reported moderately higher serum NGAL concentration without any correlation with Duke’s or T stage [31]. At the opposite, Marti et al. evidenced a 145- and 185-fold increase in serum NGAL in non-metastatic and metastatic CRC respectively, compared to controls [30].

NGAL is able to complex with MMP-9, protecting MMP-9 from its autodegradation and consequently resulting in a higher gelatinolytic action of MMP-9 on extracellular matrix. By this way, MMP-9 may promote cancer development [32, 33]. Thus, serum NGAL/MMP-9 complex could also be a marker of CRC diagnosis and/or severity.

The mechanisms underlying the increase in NGAL expression in CRC are not fully understood, but this increase could be linked to inflammation. Thus, other inflammatory serum proteins may also be potential markers for the diagnosis of CRC. While serum C-reactive protein (CRP) concentration has been shown to be higher in CRC patients than in controls in previous studies [34–36], markers such as soluble Tumor Necrosis Factor-α Receptor-1 (sTNFR-1) and sTNFR-2 have never been studied.

Thus, the main aim of the present study was to assess, in a large set of patients, the clinical value of serum levels of NGAL, NGAL/MMP-9, CRP, sTNFR-1 and sTNFR-2, for early diagnosis of CRC. Secondary aims were to explore the inter-relationships between these potential biomarkers and to examine their associations with tumoral characteristics. This analysis was based on the clinical and biological data collected in the AGARIC (**A**cides **G**ras polyinsaturés, métabolisme du tissue **A**dipeux et **RI**sque de cancer **C**olorectal) case–control study which was primarily designed to assess the role of the fatty acid composition of adipose tissue and erythrocyte membranes in CRC occurrence.

## Methods

### Study population

The AGARIC case–control study was conducted in digestive surgery departments from five University hospitals localized in north-eastern France (Besançon, Dijon, Nancy, Reims and Strasbourg) between June 2008 and June 2011. Cases were patients aged 45 years or over consecutively admitted for elective surgery with curative intent for a newly diagnosed primary CRC. CRC patients were excluded if distant synchronous metastases were known before surgery (except for *a priori* resectable liver metastases), or if they had known familial adenomatous polyposis or hereditary non polyposis CRC. However, patients with distant metastases discovered during surgery or the immediate postoperative period were kept in the analysis. Patients were also excluded if they had undergone pre-operative radiotherapy or chemotherapy in order to avoid any treatment impact on biomarkers. Control patients were aged 45 years or over, admitted in the department for elective abdominal surgery for benign diseases. They had to be free of any history of CRC or polyp resection. Cases and control patients with a history of inflammatory bowel disease, or with another malignancy in progression were excluded. In the frame of the primary purpose of the AGARIC study in the field of nutrition, cases and control patients who displayed significant changes in their dietary habits within the last three months were not considered eligible. Furthermore, patients who had a serious concomitant organic or psychic disorder that would prevent the understanding of study protocol were also excluded. A total of 224 patients with CRC and 252 controls (105 patients with hiatus or inguinal hernia, 75 patients with incisional hernia, 45 patients with diverticulitis and 27 patients with other benign illnesses) fulfilled inclusion criteria.

The study was carried out in accordance with the principles of the Declaration of Helsinki. The protocol was approved by the local Ethics Committee (CPP Est 1, Dijon, France) of the coordinating centre (date of approval: February 21st 2008) and the National Commission for Data Processing and Liberties (CNIL; date of approval: May 5th 2008). According to French law, no approval by local ethics Committee of other study centres is required. All patients signed written informed consent before their inclusion in the study. The study was registered on ClinicalTrials.gov (study NCT01966081; date of registration: October 16th 2013.

### Collection of clinical data

In each study center, clinical data were collected by the medical staff with the help of research assistants. Information about tumor characteristics was recorded from surgical and pathological reports and included the cancer site, invasion depth (pathologic T factor of the American Joint Committee on cancer (AJCC) TNM classification), maximal tumor size, lymph node metastasis and distant metastasis. Pre-surgical treatment (transfusion, anticoagulant drugs, antiplatelet drugs) was also recorded. Measurements in the pre-operative period included systolic and diastolic blood pressure, weight, waist and hip circumferences assessed in standing patients breathing normally. Height and weight history was reported by patients. Body mass index (BMI) was defined as weight (kg) divided by height squared (m^2^). In each center, cases and controls were interviewed by the same research assistant about their personal medical history, family history of CRC among first-degree relatives, marital status, education level, smoking and alcohol consumption. Medications and dietary supplements in the month preceding the inclusion were obtained from anaesthetic reports and physician prescriptions, completed by the patient’s interview. Type 2 diabetes was defined as self-reported physician diagnosis of diabetes or fasting plasma glucose ≥ 126 mg/dL or current treatment for diabetes. Leisure time physical activity (walking, biking, sports activities, do-it-yourself activities, gardening…) was defined as high if patients had an intensive physical activity (leading to sweating and/or breathlessness) > 2 hours per week, as low if patients had no intensive physical activity and a moderate activity (not leading to sweating nor breathlessness) <1 hour per week, and as medium in other situations. Alcohol intake was classified in three categories according to the approximate median in drinkers: no alcohol intake, < 5 drinks per week (reference category) and ≥ 5 drinks per week.

### Biological measurements

Pre-operative blood samples (15 mL) were collected after overnight fasting. Blood samples were immediately stored at +4°C, processed in each study center and frozen at −80°C within a maximal 4 hours. They were later transported to the coordinating center in Dijon (France) and then sent to the central biological laboratories (Inserm UMR 866, Dijon, France) or stored at −80°C according to French rules (cryopreservation in the Center of Biological Resources Ferdinand Cabanne of the Dijon University Hospital, France).

Glycemia and albumin were measured on a Vista Dimension analyzer (Siemens Healthcare Diagnostics, Deerfield USA) with the dedicated reagents (hexokinase and bromocresol purple method, respectively). Ultrasensitive CRP, albumin and prealbumin were quantified by immunonephelemetry using the same analyzer. Insulin was quantified by a chemiluminesent method on an Immulite analyzer (Siemens). NGAL, NGAL/MMP-9, sTNFR-1, sTNFR-2 were quantified by enzyme immunoassay kits (Quantikine R&D, Systems, Minneapolis, Minn). For these 3 parameters, intra and inter assay coefficients of variation were below 5 and 7%, respectively. Controls and cases were mixed on each plate and all analyses were blinded.

The homeostatic model assessment of insulin resistance (HOMA-IR) was calculated as fasting glucose (mmol/L) × fasting insulin (mU/L)/22.5.

### Statistical methods

Descriptive characteristics were expressed as percentages for categorical variables or medians with interquartile range (IQR) for continuous variables. Univariate comparisons between groups (cases and controls) were performed using chi-square tests or Fisher exact tests, when appropriate, for qualitative variables, and using the Kruskal-Wallis test for quantitative variables. Spearman correlation coefficient was used to assess correlations between quantitative variables. The ability of serum NGAL, NGAL/MMP-9 and other biological parameters (sTNFR-1, sTNFR-2, CRP) to discriminate cancer and control patients was evaluated using Receiver Operating Characteristic (ROC) curve analysis. The area under the curve (AUC) and 95% confidence interval were used to assess the discriminatory power of each biological parameter with an AUC of 1 considered perfect and 0.5 considered equal to chance. The sensitivity and specificity of each biomarker was estimated at the optimal cutoff value defined by the Youden Index [37].

Waist and/or hip circumferences were not available for 11% of the patients. Missing values were handled separately for men and women using conditional mean imputation by linear regression with age, smoking status, alcohol consumption, systolic blood pressure, HOMA-IR, recent weight-loss and BMI as predictor covariates [38]. The distribution of waist/hip ratio (WHR) and its correlations with other variables were preserved after imputation.

Unconditional logistic regression analyses stratified on center were performed for NGAL, NGAL/MMP-9, sTNFR-1, and sTNFR-2. Tertile cutpoints were determined by the distribution of each biological parameter among controls and the lowest tertile was used as the reference category. Odds ratios and 95% confidence intervals (CI) were calculated to estimate the relative risk associated with tertiles of biological parameters. Models were systematically adjusted for age, gender, body mass index, WHR, family history of CRC and alcohol intake which are known risk factors of CRC. Backward selection with p-value < 0.10 to stay in the model was performed for other patient characteristics associated with CRC with a p-value < 0.20 in the univariate analysis. Pre-albumin, recent weight-loss and regular statin use in the last month were retained in all models whereas diabetes, albumin and aspirin/non steroidal anti-inflammatory drugs (NSAIDs) use were rejected. Selected models were adjusted thereafter for CRP. Given that we were especially interested in the independent association between serum NGAL and CRC, serum sTNFR-1 and sTNFR-2 were also separately introduced into the regression model. The Hosmer-Lemeshow test was used to check models’ goodness-of-fit.

Determinants of serum NGAL, sTNFR-1 and sTNFR-2 among CRC patients were studied in a multivariate linear regression analyses systematically adjusted for age and gender. All other variables associated with serum biomarkers in univariate analysis at p < 0.10 were introduced into the regression model as independent variables. Backward selection procedure was used with p-value < 0.10 to stay in the model. The selected model was adjusted for CRP thereafter.

The significance level was p < 0.05. The statistical analyses were performed with SAS software version 9.3 (SAS Institute Inc, Cary, NC).

## Results

### Comparison of patients with colorectal cancer and control patients

As indicated in Table 1, patients with CRC were significantly older (p = 0.04) than control patients. As expected in this case–control study, CRC patients had a lower median BMI (p = 0.05) than controls, experienced more frequently a weight-loss >5 kg in the last 3 months (p < 0.001) and showed lower blood levels of pre-albumin (p < 0.001). Family history of CRC (p = 0.06), high alcohol consumption (p = 0.08), type 2 diabetes (p = 0.09) and statin use (p = 0.09) tended to be more frequent in cases than in controls but not significantly so. No significant differences in gender distribution, WHR, HOMA-IR, smoking habits, physical activity and use of aspirin/NSAIDS were observed between cases and controls.

**Table 1:** **Main characteristics of colorectal cancer cases and controls**

	Controls (n = 252)	Cases (n = 224)	P value
	n (%)	n (%)	
Male gender	144 (57)	137 (61)	0.37
Median age (years) [IQR]	66.4 [58.4-74.8]	69.6 [60.6-75.9]	0.04
Median current BMI (kg/m^2^) [IQR]	26.9 [24.2-30.4]	25.9 [23.3-29.8]	0.05
Median Waist/Hip ratio [IQR]	0.93 [0.88-1.00]	0.95 [0.89-1.01]	0.12
Recent weight loss ≥5 kg	30 (12)	53 (24)	<0.001
Severe denutrition	6 (2)	15 (7)	0.02
Family history of colorectal cancer			0.06
No	220 (87)	178 (79)	
Yes	22 (9)	29 (13)	
Unknown	10 (4)	17 (8)	
Smoking			0.72
Never smoker	113 (45)	107 (48)	
Ex smoker	103 (41)	89 (40)	
Current smoker	37 (15)	28 (13)	
Alcohol consumption			0.08
None	47 (19)	39 (18)	
<5 drinks/week	105 (42)	74 (33)	
≥5 drinks/week	98 (39)	109 (49)	
Physical activity			0.29
Low	65 (26)	70 (31)	
Medium	139 (55)	108 (48)	
High	48 (19)	46 (21)	
Type 2 diabetes	40 (16)	49 (22)	0.09
Median HOMA-IR	0.81 [0.42-1.98]	0.92 [0.44-2.15]	0.28
Median pre-albumin (g/L)	0.26 [0.22-0.30]	0.23 [0.19-0.28]	<0.001
Aspirin or NSAID use in the last month	51 (20)	52 (23)	0.43
Statin use in the last month	60 (24)	m 69 (31)	0.09
TNM stage			
(Tis)		13 (5.8)	
Stage I		57 (25.4)	
Stage II		77 (34.4)	
Stage III		60 (27.1)	
Stage IV/unstaged		17 (7.6)	

**Figure 1:**
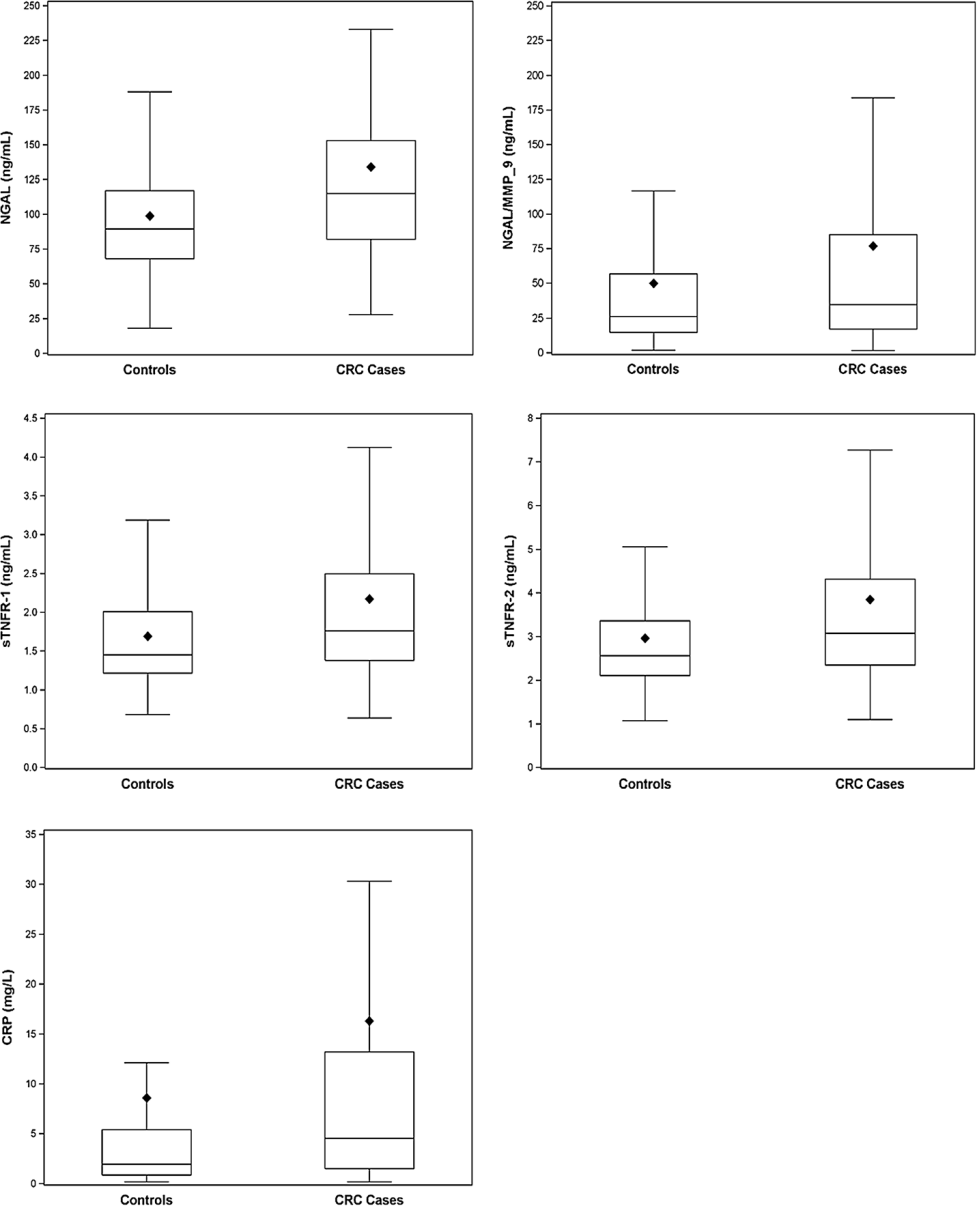
**Serum levels of NGAL, NGAL/MMP-9, sTNFR-1, sTNFR-2 and CRP in colorectal cancer cases and controls.** The boundary of the box closest to zero indicates the 25th percentile, a line within the box marks the median, and the boundary of the box farthest from zero indicates the 75th percentile. The lozenge symbol indicates the mean. Error bars above the boxes indicate the maximum observation above the third quintile (Q3) plus 1.5 multiplied by the difference between the values of the third and the first quintile (Q3-Q1). Errors bars below the boxes indicate the minimum observation below Q1 minus 1.5× (Q3-Q1). All p values for differences between CRC cases and controls were <0.001 using Kruskal-Wallis test.

In univariate analyses, higher serum concentrations of NGAL, NGAL/MMP-9, CRP, sTNFR-1, sTNFR-2 (all p values <0.005) were observed in patients with CRC compared to controls (Figure 1). Among cases and controls, median levels (interquartile range: IQR) were respectively 115 ng/mL (82–153) *versus* 89.5 (68–117) for NGAL, 35.0 ng/mL (17.1-85-5) versus 26.3 (15.0-57.1) for NGAL/MMP-9, 4.54 mg/L (1.49-13.2) *versus* 1.96 (0.56-5.39) for CRP, 1.76 ng/mL (1.38-2.50) *versus* 1.45 (1.22-2.01) for sTNFR-1 and 3.07 ng/mL (2.35-4.32) *versus* 2.56 (2.11-3.36) for sTNFR-2. These results were virtually unchanged after exclusion of control patients with diverticulitis. The ROC analysis revealed that the discriminative power of serum NGAL between CRC patients and controls was moderate as assessed by an area under the curve (AUC) of 0.65 (95% CI:0.60-0.70) and poor for NGAL/MMP-9 with an AUC of 0.58 (95% CI:0.52-0.63). The discriminative power of other biological parameters was not better with an AUC of 0.64 (95% CI:0.59-0.69) for sTNFR-1, 0.63 (95% CI:0.58-0.68) for sTNFR-2, and 0.63 (95% CI:0.58-0.68) for CRP. For the optimum cutoff value of NGAL (>106 ng/mL) and NGAL/MMP-9 (>71.7 ng/mL), sensitivites and specificities were respectively 57 and 69% for NGAL and 34 and 81% for NGAL/MMP-9. The optimum cutoff values of sTNFR-1 (>1.56 ng/mL), sTNFR-2 (>3.58 ng/mL) and CRP (>4.45 mg/L) provide sensitivities and specificities of 66 and 58%, 41 and 79%, 52 and 71%, for each biomarker respectively. For all biological parameters, the exclusion of control patients with diverticulitis had only a marginal impact on AUC values.

Among cases, there were strong inter-relationships between NGAL, sTNFR-1, sTNFR-2, and CRP (Table 2). Spearman correlation coefficients were especially high between serum NGAL and both sTNFR-1 and sTNFR-2 (p < 0.001); they were lower with CRP. Although the association between NGAL and NGAL/MMP-9 was relatively high (p < 0.001), correlation coefficients between the complex and other serum markers were very low. Among controls, associations between NGAL and other serum markers were attenuated for sTNFR-1, and sTNFR-2, although being highly significant (p < 0.001). In controls, NGAL/MMP-9 remained highly correlated to NGAL (p < 0.001) and weakly associated with other serum markers (CRP, sTNFR-1, sTNFR-2).

**Table 2:** **Spearman correlation coefficients between NGAL and other inflammatory markers in colorectal cancer cases and controls**

	CRP	NGAL	NGAL/MMP-9	sTNFR-1	sTNFR-2
CRP	1	*0.14* ^*a*^	*0.19* ^*b*^	*0.43* ^*c*^	*0.35* ^*c*^
NGAL	**0.41** ^**c**^	**1**	*0.41* ^*c*^	*0.43* ^*c*^	*0.44* ^*c*^
NGAL/MMP-9	**0.15** ^**a**^	**0.46** ^**c**^	1	*0.15* ^*a*^	*0.06*
sTNFR-1	**0.51** ^**c**^	**0.65** ^**c**^	**0.15** ^**a**^	1	*0.78* ^*c*^
sTNFR-2	**0.42** ^**c**^	**0.59** ^**c**^	**0.04**	**0.85** ^**b**^	1

^a^p < 0.05, ^b^p < 0.01, ^c^p < 0.001.

Correlation coefficients are indicated in bold characters for cases (n = 219) and in italics for controls (n = 250).

Multiple logistic regression models were used to test the associations between serum NGAL, NGAL/MMP-9, sTNFR-1, sTNFR-2, and the risk of CRC. After adjustment for known risk factors of CRC (age, gender, BMI, WHR, family history of CRC, alcohol intake) and for statin use, recent weight loss and serum pre-albumin, we observed a significant association between serum NGAL and the risk of CRC (3rd *versus* 1st tertile, OR: 2.76; 95% CI:1.59-4.78) (Table 3). Significant associations with the risk of CRC were also found for sTNFR-1 and sTNFR-2 but not with NGAL/MMP-9. Further adjustment for CRP did not affect these associations (Table 3). When sTNFR-1 and sTNFR-2 were each concurrently considered with serum NGAL in the regression model, none of them could enter the model with p values of 0.14 and 0.52 respectively.

**Table 3:** **Multivariate associations between serum levels of NGAL, sTNFR-1, sTNFR-2 and risk of colorectal cancer**

		Multivariate logistic model ^a^			Multivariate logistic model adjusted for CRP ^b^		
Serum marker (ng/ml)	Number of case/controls	OR	95% CI	P value ^c^	OR	95% CI	P value ^c^
NGAL				<0.001			<0.001
< 75	40/82	1			1		
75- 104	52/85	1.42	0.81-2.50		1.43	0.81-2.50	
≥ 104	127/83	2.76	1.59-4.78		2.81	1.61-4.89	
NGAL/MMP-9				0.75			0.74
< 18.5	60/83	1			1		
18.5-42.3	61/84	0.95	0.56-1.62		0.95	0.56-1.62	
≥ 42.3	98/84	1.16	0.65-2.06		1.17	0.66-2.09	
sTNFR-1				0.015			0.013
< 1.30	38/83	1			1		
1.30-1.77	72/84	2.00	1.14-3.50		2.03	1.16-3.55	
≥ 1.77	109/83	2.44	1.34-4.45		2.51	1.36-4.61	
sTNFR-2				0.016			0.015
< 2.23	47/83	1			1		
2.23-3.09	63/85	am 1.34	0.79-2.28		am 1.34	0.79-2.29	
≥ 3.09	109/82	1.93	1.12-3.31		1.95	1.13-3.37	

^a^Models were stratified on centre and adjusted for age, gender, body mass index, waist/hip ratio, family history of colorectal cancer, alcohol intake, pre-albumin, recent weight-loss and regular statin intake in the last month.

^b^C reactive protein (CRP) was forced in the multivariate models (p values for association with the risk of colorectal cancer were respectively of 0.58, 0.87, 0.60, 0.69 in the NGAL, NGAL/MMP-9, sTNFR-1 and sTNFR-2 models).

^c^p value for trend.

### Serum levels of NGAL, sTNFR-1 and sTNFR-2 according to tumor characteristics

**Figure 2:**
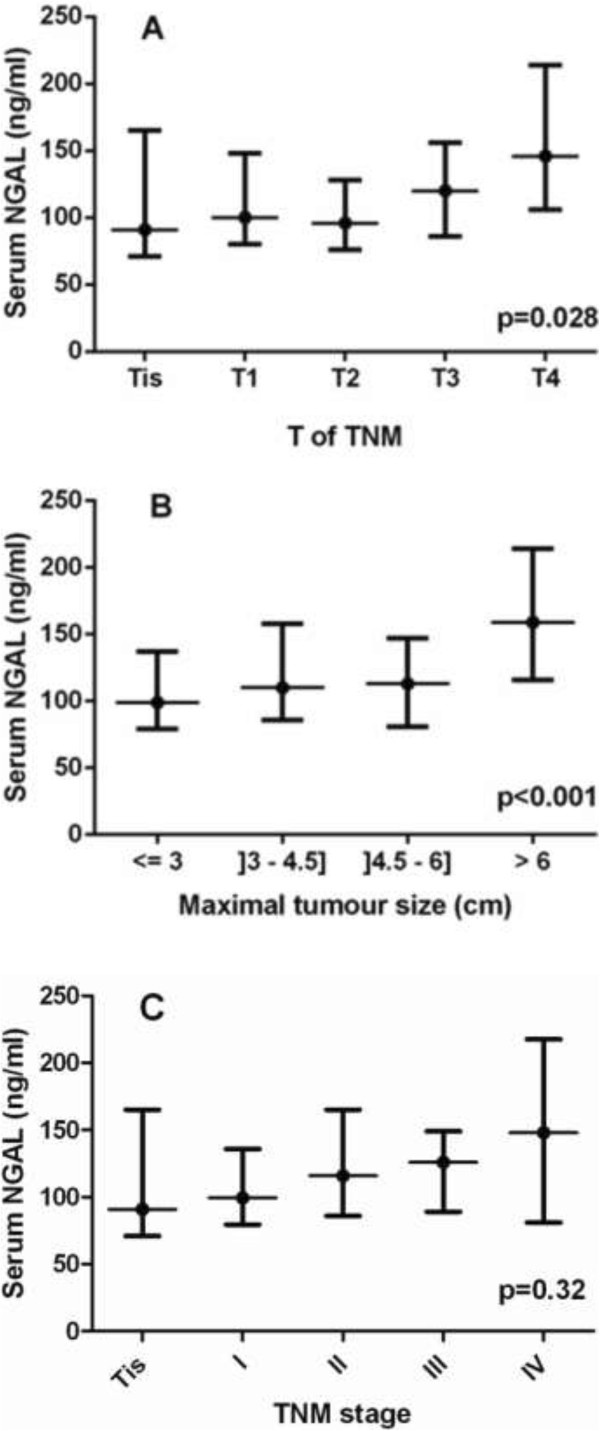
**Serum NGAL concentrations according to invasion depth (Panel A), maximal tumoral size (Panel B) and TNM stage (Panel C) of colorectal cancer.** Values are presented as medians and interquartile ranges. Maximal tumor size was unknown in 9 patients with CRC and blood samples were missing in 5 cases.

Tumors were located in the rectum for 48 patients (21%), left colon for 85 (38%), right colon for 83 (37%) and 8 patients presented multiple locations (4%). There were no significant differences in biological parameters by location. The distribution of TNM stages given in Table 1 showed that only 34% of the patients had advanced CRC (stages III and IV/unstaged). As indicated in Figure 2, serum NGAL levels significantly increased with the invasion depth (p = 0.028) with median values (IQR) ranging from 91 ng/mL (71–165) for Tis tumors, to 100 ng/mL (80–148) for pT1, 96 ng/mL (76–128) for pT2, 120 ng/ml (86–156) for pT3 and 146 ng/mL (106–214) for pT4. Serum NGAL was also significantly associated with maximal tumor size (p < 0.001) with median values (IQR) ranging from 99 ng/ml (79–137) for tumors ≤3 cm, to 110 ng/ml (86–158) for tumors between 3 and 4.5 cm, 113 ng/mL (81–147) for tumors between 4.5 and 6 cm and 159 ng/mL (116–214) for tumors > 6 cm. We did not observe any significant association between NGAL levels and lymph node involvement (p = 0.65), distant metastasis (p = 0.17) and TNM stage (p = 0.32). Serum levels of sTNFR-1 tended to be associated with maximal tumor size (p = 0.064) and lymph node involvement (p = 0.056) whereas serum sTNFR-2 levels were only associated with maximal tumor size (p = 0.046). No associations were found between serum NGAL/MMP-9 and tumor characteristics or stage (all p values >0.30).

### Independent determinants of serum levels of NGAL, sTNFR-1 and sTNFR-2

Multivariate linear regression analysis showed that, among CRC patients, serum levels of NGAL significantly decreased with serum albumin (p < 0.001) and were positively associated with WHR (p = 0.005), recent weight loss >5 kg (p = 0.026) and maximal tumor size (p = 0.028) (Table 4). Past and current smoking, age and male gender were not independently associated with serum levels of NGAL. The introduction of serum CRP in the regression model showed that CRP was an important significant determinant of serum NGAL (p = 0.004) which reduced the effects of other variables. Especially, the effect of tumor size on serum NGAL was reduced by 26% and no longer reached the significance level (p = 0.11). None of the tumoral characteristics were independently associated with serum levels of sTNFR-1 and sTNFR-2. Both inflammatory markers were independently related to increasing age (p = 0.007 and p = 0.021 respectively), waist/hip ratio (p = 0.004 and p = 0.019 respectively) and to decreasing albumin levels (p = 0.037 and p = 0.019, respectively). Serum CRP was also a strong determinant of sTNFR-1 levels (p < 0.001), but less strongly related to sTNFR-2 levels (p = 0.058). Last, high physical activity was only associated with decreasing sTNFR-2 levels (p = 0.012). The sole independent determinants of NGAL/MMP-9 were CRP (p = 0.03) and current smoking (p < 0.001).

**Table 4:** **Independent determinants of serum NGAL among patients with colorectal cancer in multiple linear regression analysis**

	Multivariate model			Multivariate model further adjusted for CRP		
	Regression coefficient	Standard error	P value	Regression coefficient	Standard error	P value
Age^a^ (for 10 year increase)	7.3	5.0	0.15	9.2	4.9	0.063
Male gender^a^	11.8	13.3	0.38	11.4	13.0	0.38
Albumin in g/L (for 1 SD increase)	−18.9	5.3	<0.001	−12.8	5.8	0.030
Waist/hip ratio (for 1 SD increase)	18.9	6.6	0.005	16.4	6.5	0.013
Past smoking	21.5	11.6	0.066	20.8	11.4	0.069
Current smoking	27.4	16.1	0.092	28.1	15.8	0.078
Recent weight-loss	27.7	12.4	0.026	20.5	12.2	0.094
Tumoral size (for 10 mm increase)	5.0	2.3	0.028	3.7	2.3	0.11
CRP in mg/L (for 1 SD increase)	-	-	-	17.2	5.8	0.004

NGAL values were expressed in ng/ml.

^a^Age and gender were forced in the multiple regression model.

Among control patients, the sole significant determinants of serum NGAL were age (p < 0.001), male gender (p = 0.021) and serum CRP (p < 0.001). The sole significant determinants of sTNFR-1 and sTNFR-2 levels were age (p < 0.001 for both markers), body mass index (p = 0.014 and p = 0.006, respectively) and CRP levels (p < 0.001 and p = 0.007, respectively).

## Discussion and conclusions

Our case–control study demonstrated a significant increase in serum NGAL levels in CRC patients compared to controls, which persisted after adjustment for traditional risk factors for CRC. However, the increase in NGAL concentration was mainly confined to CRC patients with large-sized tumors or important depth invasion and was not observed in patients with lymph node, distant metastases and TNM stage. These findings along with the moderate discriminative power of serum NGAL suggest that, although serum NGAL may have a potential value for the evaluation of parietal invasion, it is not a suitable biomarker for diagnosis of CRC. At the optimal NGAL cutoff of 1.06 ng/mL, our estimates of sensitivity and specificity indicated that more than 40% of CRC patients would not be diagnosed whereas more than 30% of individuals would be falsely suspected of CRC.

In our study, the increase in serum NGAL concentration in patients with CRC was moderate since NGAL concentration was on average 28% higher in CRC patients than in controls, varying between 11% in stage 0-I and 66% in stage IV. Our results along with those reported by other studies both in the oncology field and in other fields [14, 21, 25, 39–41] contrast with the recent observation of 145- and 185-fold increases in serum NGAL in non-metastatic and metastatic CRC respectively, compared to controls [30]. Besides differences in study design, the lower increase in serum NGAL in our study could be partly explained by differences in NGAL measurement techniques. We used a method calibrated with human recombinant lipocalin-2 which is considered as specific by the manufacturer. However, both studies concur regarding the positive association between serum NGAL levels and tumor invasion depth and the lack of association with lymph node status.

Our results also differ from those reported by Sun et al. [27], who did not show any significant increase in serum NGAL concentration in 39 CRC patients compared to matched controls, nor any association with cancer stage. However, the previous study observed similar trends to ours, which could have been possibly stronger with a larger cohort. Furthermore, the different findings relative to serum NGAL markedly contrast with the clear demonstration that NGAL expression gradually increases along the adenoma-carcinoma sequence in 526 specimens of colorectal tissue [27]. The absence of NGAL expression was observed in 80.9% of histologically normal mucosa specimens, 56.2% and 37.5% of adenomas with low-grade and high-grade dysplasia respectively, and in only 5.9% of carcinoma specimens. In addition, NGAL expression was associated with cancer stage and tumor recurrence in stage II cancer patients. Mc Lean et al. reported similar results, showing that 100% of cancer lesions and 67% of adenomas expressed NGAL in patients with CRC and colonic adenoma, respectively [41]. In this study, serum NGAL concentration was not measured.

At the opposite, it has been demonstrated on a preclinical model of colon carcinogenesis that NGAL was upregulated only in advanced stages of tumor progression. This is relatively concordant with the present results that showed a link between the serum concentration of NGAL in CRC patients and invasion depth [42]. On the other hand, in a recent review, Candido et al. concluded that NGAL was upregulated in adenocarcinoma tumor samples but that its expression was reduced in metastatic samples [32]. This conclusion is in agreement with the lack of marked difference in NGAL concentration in patients with or without lymph node or metastasis.

Altogether, our results and those of previous studies support the hypothesis that NGAL could contribute to colorectal carcinogenesis [27, 30]. However, there is no clear evidence that serum NGAL could be a robust marker for CRC diagnosis as reflected by its modest discriminative power between cases and controls. This may be due to the fact that NGAL is not specifically secreted by cancer cells and may be produced by numerous tissues in response to stress conditions, especially inflammatory states. Besides adenocarcinoma, NGAL is also overexpressed in inflammatory bowel diseases and diverticulis [43]. Our multivariate analysis of determinants of serum NGAL showed that NGAL concentration was negatively correlated with albumin and positively with WHR, CRP and tumoral size, each of these parameters being known to be associated with inflammation. Adjustment for CRP reduced, but not totally abolished, the association between serum NGAL and tumoral size. In the lack of data about tumoral expression of NGAL in our study, the possible role of inflammation for explaining high NGAL concentrations in CRC remains speculative. Increased serum NGAL may be simply due to a global systemic response to the presence of cancer as reflected by high CRP levels in CRC patients. Alternatively, NGAL may be secreted by tumoral cells either under the influence of local peritumoral inflammation or under the influence of both inflammatory cytokines and other factors not yet identified.

The association of NGAL with MMP-9 protects MMP-9 from its autodegradation and increases the gelatinolytic action of MMP-9 on extracellular matrix. It has been suggested that MMP-9 may promote cancer development by this way [32, 33]. On the other hand, NGAL can also promote cell motility and invasion of colon carcinoma cells, in a MMP9-independent manner [44]. Serum concentration of the NGAL/MMP-9 complex was moderately higher in our CRC patients than in controls but its discriminative power was poor, meaning that it can not be used as a diagnosis marker for CRC. Moreover, no associations were found between serum NGAL/MMP-9 and tumor characteristics including depth invasion or stage. Our results must be interpreted with caution because they have been obtained in serum samples, but they do not support any important role for NGAL/MMP-9 complex in CRC development.

There is some evidence that NGAL expression can be induced by several cytokines and growth factors [45–47]. Our study showed strong relationships between serum levels of NGAL and other inflammatory markers such as sTNFR-1, sTNFR-2 and CRP both in CRC and control patients. However, these associations were stronger in CRC patients than in controls, further suggesting that inflammation due to cancer could play a predominant role. We extended previous findings relative to CRP [34–36] by showing that, not only serum CRP, but also serum levels of both sTNFR-1, sTNFR-2 were higher in CRC patients than in controls even after adjustment for risk factors of CRC. This can be explained by the fact that sTNFR-1 is expressed ubiquitously and can be overexpressed by epithelial cancer cells, whereas sTNFR-2 is predominantly expressed by lymphoid cells in particular by those that infiltrate the tumor. Very few clinical or epidemiological studies have been published regarding these biomarkers. Only two previous nested case–control studies examined the prospective association between plasma levels of sTNFR-2 and the risk of CRC and provided inconsistent results in men and women [48, 49]. To our knowledge, serum levels of sTNFR-1 and sTNFR-2 have never been studied as potential diagnostic biomarkers of CRC. Despite the significant increase in serum sTNFR-1 and sTNFR-2 in CRC patients, the intensity of their association with CRC was lower than that observed with serum NGAL. Furthermore, both markers had a poorer discriminative power than serum NGAL, and only presented marginal associations with tumoral size and lymph node involvement. These observations do not support the idea that serum sTNFR-1 and sTNFR-2 could be of interest for helping diagnosis of CRC.

Our study presented some limitations. First, the cross-sectional design precludes any temporal inference being made and any causal conclusion being drawn from our results. Second, this study may have been subject to selection biases. CRC patients recruited in university hospitals may not be representative of all CRC and, because patients with pre-operative treatment were discarded for other study purposes, relatively few patients with rectal cancer were included. However, our study did not provide any evidence that serum biomarkers depended on primary cancer location. The recruitment of control patients operated for benign diseases with possible inflammatory background could have reduced the effect of potential biomarkers. However, our results were not altered after exclusion of such controls. On the other hand, our study had the advantage of including large samples of well characterized cases and controls which allowed potential confounders to be taken into account, and of relying on centralized and blinded analysis of blood samples stored in optimal conditions. Finally, we did not compare NGAL and sTNFRs with usual tumoral markers such as CEA, CA19-9. Such a comparison could have been interesting if the markers we quantified had demonstrated their potential usefulness in routine but has a lesser interest in front of the results we observed.

In conclusion, this case–control study showed that NGAL, NGAL/MMP9, sTNFR-1 and sTNFR-2 serum concentrations are higher in patients with CRC, and that NGAL levels are especially elevated in patients with large tumors. However, our findings do not suggest that these serum parameters may be clinically relevant markers for the detection of CRC and especially for early detection.

## Abbreviations

AUC: Area under the curve
BMI: Body mass index
CI: Confidence interval
CRC: Colorectal cancer
CRP: C reactive protein
HOMA-IR: Homeostatic model assessment of insulin resistance
IQR: Interquartile range
MMP9: Matrix metallopeptidase 9
NGAL: Neutrophil gelatinase-associated lipocalin
NSAID: Non steroidal anti-inflammatory drugs
OR: Odds ratio
ROC: Receiver operating characteristic
sTNFR: Soluble tumor necrosis factor receptor 1 and 2.
